# Movement Dynamics and Habitat Use of Owned and Unowned Free-Roaming Dogs on a Two-Square-Kilometer Tourist Island in Southern Thailand

**DOI:** 10.3390/vetsci12121181

**Published:** 2025-12-10

**Authors:** Thanidtha Te-Chaniyom, Kittisakdi Choomalee, Kyaw Ko Ko Htet, Anuwat Wiratsudakul, Virasakdi Chongsuvivatwong

**Affiliations:** 1 Department of Epidemiology, Faculty of Medicine, Prince of Songkla University, Songkhla 90110, Thailand; thanidtha.t@psu.ac.th (T.T.-C.); kyawkkhtet@gmail.com (K.K.K.H.); 2 Faculty of Veterinary Science, Prince of Songkla University, Songkhla 90110, Thailand; 3 Department of Family and Preventive Medicine, Faculty of Medicine, Prince of Songkla University, Songkhla 90110, Thailand; kittisak.c@psu.ac.th; 4 The Monitoring and Surveillance Center for Zoonotic Diseases in Wildlife and Exotic Animals, Faculty of Veterinary Science, Mahidol University, Nakhon Pathom 73170, Thailand; anuwat.wir@mahidol.ac.th; 5 Department of Clinical Sciences and Public Health, Faculty of Veterinary Science, Mahidol University, Nakhon Pathom 73170, Thailand

**Keywords:** free-roaming dogs, movement pattern, habitat land type preferences, tourist, Thailand

## Abstract

Free-roaming dogs are a neglected population that poses public health concerns in tourist areas. This study, conducted during both the high and low tourist seasons, aimed to (i) investigate the movement patterns of free-roaming dogs on the Lipe tourist destination island, southern Thailand, focusing on their daily distance, and (ii) identify habitat land type preferences. Between September and December 2024, eight owned and seven unowned dogs were fitted with Catlog GPS devices. Tracking data were collected for the dog’s coordinates every 15 min for at least four consecutive days. Data analysis and visualization were performed using R software. On average, dogs traveled 5300 m daily. Unowned dogs exhibited their highest speed on beaches (median = 70, IQR = 44–79 m per 15 min). Free-roaming dogs spent most of their time in human habitats. The time of day influenced dogs’ appearance across different land types. In conclusion, free-roaming dogs on the island traveled longer distances than in most previous studies. The dogs mostly preferred being around human habitats. Land types influenced dogs’ speed. Dogs appeared on different land types at different times of day. Guided by these data, we recommend (i) better dog registration and other measures to reduce free-roaming activities of dogs and (ii) regular blanket coverage of dog rabies immunization to make the island safer for tourists and residents.

## Simple Summary

On this 2 km^2^ island, the dog samples traveled an average of around 5000 m per day and spent 75% of their time near human habitats. Unowned dogs spent around one-third of their time in the forest. These findings suggest the island is vulnerable to rapid transmission of dog-related diseases. Measures to achieve a high immunity level of rabies throughout the entire year, as well as restrictions on dog-roaming on the island, are needed.

## 1. Introduction

### 1.1. Free-Roaming Dogs

Dogs have coexisted with humans for centuries, making them one of the most popular companion animals in many cultures worldwide [1]. However, there are differences in the norms of ownership between Western and Eastern countries, including welfare and regulations [2,3]. Although regulations of confinement and leashing in public areas exist in many Western countries, the vast majority of dogs, both owned and unowned, are free-roaming in Eastern and low- and middle-income countries (LMIC), including Southeast Asia and Thailand [4,5,6]. These are areas where dog rabies is endemic [5,7,8,9].

### 1.2. Importance of Free-Roaming Dogs in Tourist Areas

The presence of free-roaming dogs in tourist areas has become a significant public health concern, including zoonotic diseases [10,11,12,13,14,15,16,17] and dog bite injuries [18,19,20]. A study from Pakistan found that most dog bite incidents involved stray dogs rather than pet dogs [20]. A significant public health challenge arises from the interaction between tourists and free-roaming dogs, which is facilitated by the generally dog-friendly attitudes of many tourists [21,22,23]. Dogs in high flux areas are generally friendly towards humans [22,24,25,26].

Rabies is an almost 100% fatal zoonotic disease that can infect all mammals [27]. Most rabies patients have a history linked to exposure to a dog [28]. A previous study of travelers from high-income countries who visited Southeast Asia reported exposure to potentially rabid animals, including being bitten and licked, with an incidence rate of rabies exposure of 34 (95% CI, 24–48) per month per 1000 travelers [29]. The fundamental problem lies in the high dog population density, driven by factors such as owner negligence, lack of sterilization, and abandonment of food sources [7,28,30]. Therefore, understanding the movement patterns and habitat preferences of free-roaming dogs is critical for effective planning of public health interventions and reducing disease burden.

### 1.3. Dog Movement Patterns

Previous studies have demonstrated a wide range of daily distances and peak times of travel of free-roaming dogs throughout the world in places such as Chile, Australia, and Taiwan [31,32,33]. Such distance implies a dog’s potential for the spread of infectious diseases among dogs, especially rabies [34]. Most free-roaming dogs average a daily travel of approximately 1 to 3 km [31,32,33]; however, in Kenya, the dogs can wander over 10 km [11].

Domestic dogs exhibit diurnal patterns [35,36]. Most studies have reported that twilight [32,37,38] and morning [32,38] are the peak times for dogs’ wandering. However, free-roaming male dogs in Chile were more frequently detected by GPS during midday, from 12:00 to 16:00 h [31]. In Southern Asia, dogs were observed to be more active in the morning and evening during a pilot study [38] (all summarized in Table S1).

### 1.4. Dogs’ Land Type Preferences

The habitats of free-roaming dogs are explained by relating them to the biological needs of the species, including access to water [39] and food resources [30,40], brushlands [41], and suitable shelters, as well as physical and mental stimulation [31]. In Malaysia, the density of free-roaming dogs was highly correlated with areas dominated by Indian ethnicities and areas with high levels of garbage [38]. Dogs without identified owners in Chile were less likely to venture into rural areas if they were located closer to the border between rural and urban areas [31]. Free-roaming, unleashed dogs in Indonesia and Guatemala, whether in urban or rural areas, preferred low-vegetation areas and buildings over roads [42].

### 1.5. The Situation in Thailand

In Thailand, dog rabies is an endemic situation [5,9]. The dog population has been estimated to be nearly 12 million [43], comprising owned and unowned dogs. It has been reported that more than 40% of owned dogs roam freely [44]. The most common places where free-roaming dogs without owners exist are villages, public spaces, temples, beaches, and main roadsides [45]. Despite the prevalence of free-roaming dogs in Thai culture, no scientific publication could be found on the movement patterns of Thai free-roaming dogs.

### 1.6. Lipe Island in Southern Thailand

Lipe Island is a small (2 km^2^) tourist destination island located in Satun Province, southern Thailand. Approximately 680,000 visitors stayed for an average of 1–3 days in 2024 [46,47]. The number of residents was reported to be around 1000 people [48].

## 2. Rationale and Objectives

Thailand is both a renowned international tourist destination and an endemic country for dog rabies, as well as a culture with a significant number of free-roaming dogs. Yet, there have been only a limited number of studies on the dynamic movement and habitation of free-roaming dogs. Lipe Island is a world-famous confined island with a high density of human and dog populations. This raises the risk of dog-related disease transmission. Therefore, the movement patterns and habitats of free-roaming dogs should be elucidated to support potential management and mitigation strategies.

To fill these knowledge gaps, we conducted this study between September and December 2024, aiming to (i) investigate the movement patterns of free-roaming dogs on Lipe Island, focusing on their daily distance covered, and (ii) identify their habitat land type preferences. These two objectives were categorized by ownership status and day–night and tourist season variation.

## 3. Methodology

### 3.1. Study Area

- Location and land types

**Figure 1:**
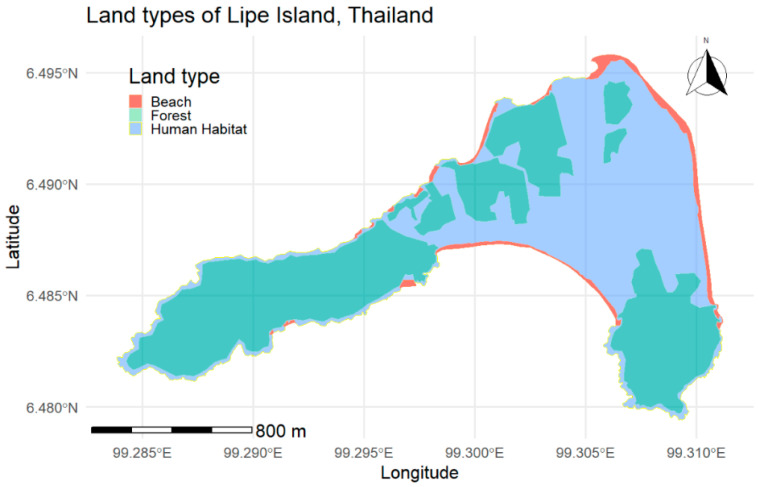
Land types comprise forest or shrub (green), beaches (orange) [49], and human habitat (blue) areas covering Lipe Island. Image modified from OSM (the OSM is licensed under the Creative Commons Attribution-Share Alike 2.0 license (CC BY-SA 2.0)).

Lipe Island is located between latitude 6°28′ and 6°35′ North and between longitude 99°09′ and 99°21′ East, situated near the border with Malaysia. The island has a diverse range of landscapes, including (a) forests and shrublands (green areas) and (b) beaches (orange areas), as shown in Figure 1, modified from the OpenStreetMap (OSM) database [49]. In addition to these, human habitat land types include residential, commercial, tourist accommodation, and roadside areas (blue areas).

- Tourist seasonality

Lipe Island has two distinct seasons: a rainy, low tourist season (June to October) and a less rainy, high tourist season (November to May). In 2024, approximately 410,000 guests visited during high tourist season, and around 270,000 guests visited during low tourist season [46,47].

- Time of day

We categorized the time of day as 04:00–09:59 for morning, 10:00–15:59 for midday, 16:00–21:59 for evening, and 22:00–03:59 for late night. This categorization was applied to analyze possible diurnal variation in the dogs’ behaviors.

- Dogs

The Regional Livestock Office 9 (RLO 9) estimated that 120 dogs were on the island during 2022, but there was no dog registration. The average dog population density was estimated to be at least 50 dogs per km^2^, which is twice the previous estimate for the country (21.7 and 3.2 head per km^2^ for owned and unowned dogs, respectively) [43].

On the island, there are owned dogs and unowned dogs. Alien dogs can visit or be introduced to the island by tourists or residents without restriction. Most owned dogs are not entirely confined to their owners’ homes/properties, which are mostly unfenced. Based on discussions with local residents, most unowned dogs are regularly fed by different groups of zoophiles who are dog lovers.

### 3.2. Study Period

Along with the tourist season, data collection was conducted during September and October 2024 for the low tourist season and during December 2024 for the high tourist season.

### 3.3. Dog Selection in This Study

We adopted the World Organization for Animal Health (WOAH) [50] definition of free-roaming dog as “owned or unowned dogs allowed to roam freely and commensally under no direct human supervision, and which retain dependence on humans.” [42]. We selected a set of dogs claimed to be owned by a group of residents on the island, while unowned dogs without any claimants were also chosen with the assistance of the zoophiles. The dog selection also considered variations in locations throughout the island.

### 3.4. Eligibility Criteria for the Study Dogs

Both sexes of healthy dogs aged 1 year or older, as estimated by the primary investigator (TT) at the time of the first visit, and with a withers height of at least 35 cm (The Kennel Club, 2018, as cited in [39]) were included. We excluded dogs that were too small, as they were outliers and not comparable in movement distance.

### 3.5. Sample Size

Using the sample size for estimating a finite population mean [51,52,53] of the daily distance of the dog in the previous study,

$$n=\frac{N\sigma^{2}z_{1-\frac{\alpha }{2}}^{2}}{d^{2}\left(N-1\right)+\sigma^{2}z_{1-\frac{\alpha }{2}}^{2}}$$

Finite population (*N*) = 120, 95% confidence interval = 1.96, standard deviation of daily distance of distance (SD) = 0.55 [33], and standard error (*d*) = 0.5 were used to calculate the sample size. The estimated sample size was five dogs per tourist season, a total of 10 across the low and high seasons. There were initially 10 GPS collars available. It was feasible to use them with 15 dogs in each tourist season, which was at least 10% of the estimated dog population on the island.

### 3.6. Dog Tracking

The primary investigator coordinated with village health volunteers to identify dog owners willing to give consent for collar fitting. Unowned dogs were identified by three zoophiles familiar with the dogs, allowing for safe approaches. Dog characteristics were recorded with verbal consent from the owner or a reachable person. Once the researcher and zoophiles selected unowned dogs, they approached the dogs and fitted the collars. The zoophiles, who regularly fed the dogs, could remove collars at any time if they sensed the dogs were uncomfortable. Finally, the researchers and zoophiles worked together, with the zoophiles removing the collars and handing them over to the researchers. The same procedures were followed by the dog owners.

Each dog was fitted with a collar containing a Catlog Gen 2 GPS device, Dallas, TX, USA weighing less than 100 g [54], which was less than 1% of the dog’s body weight. The device was already housed in plastic cases measuring 38 × 32 × 17 mm fixed onto nylon collars (Figure S1). GPS data were automatically recorded every 15 min for at least four to seven consecutive days, chosen to balance battery life and capture movement transitions [32]. These GPS devices had been tested for precision before being fitted on the dogs. The median (IQR) values of the recorded distance between two readings 15 min apart on the same fixed points were 28 m (IQR: 15–50 m), which was considered acceptable in this setting. After collecting the collar, the researchers provided deworming medication to the owners or the responsible caregiver for the animal’s welfare.

### 3.7. Data Management

After removing the collar with the GPS device, the GPS-fixed data, including latitude, longitude, date, time, and number of satellites, as well as horizontal dilution of precision (HDOP), were converted into CSV format using the CatLog GPS Control Center v4.1 software (https://www.mr-lee.com/catlog.htm (accessed on 3 December 2025)). Data points were excluded if HDOP > 5 [55] or if their coordinates were outside Lipe Island. The distance between two points within the 15 min intervals was computed using a formula applied from trigonometry theory [56]. Time points with missing GPS coordinates resulted in the exclusion of the corresponding travel distance (i.e., for the interval immediately before and after the missing point). When the time interval between two points exceeded 25 min, the distance was also considered missing. The flow of the data cleaning process is in Figure S1.

Lipe Island map was featured as forests or shrubs (“forests” from hereon), and beaches were modified from the OpenStreetMap (OSM) database [49]. Other areas were defined as human habitats by the researchers for the purpose of the study. Each coordinate from the collar-based recording was identified as a land type. When calculating movement speed using two location points that fall within different land types, the preceding land types were used to determine the locations. All data from GPS devices were managed and analyzed using R version 4.5.0 [57].

### 3.8. Data Analysis

**Figure 2:**
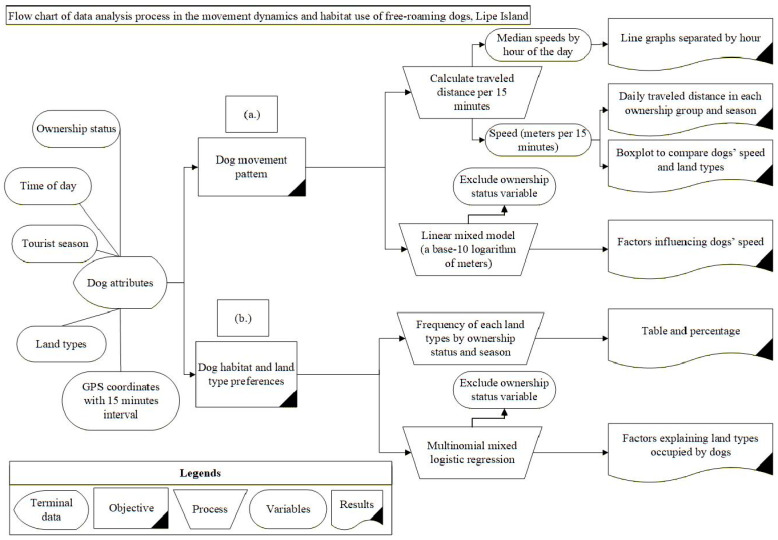
Data analysis process for the movement dynamics and habitat use of free-roaming dogs, Lipe Island.

Data analysis was conducted in two separate parts according to the study objectives. The analysis process shown in Figure 2 describes the dogs’ movement patterns (in part a) and identifies the dogs’ habitat and land type preferences (in part b).

- Dogs’ movement patterns (a)

The dogs’ daily traveled distance was compiled from the distance between two adjacent time points over the whole day (4 intervals × 24 h). The dogs’ speed, measured as the travel distance per 15 min, underwent descriptive analysis. The median and interquartile range (IQR) of dog speed were calculated for the land types. The variation in the dog’s speed was displayed as an hourly travel distance around the clock.

A linear mixed model (LMM) was employed to identify the factors influencing the dogs’ speed. To reduce the influence of outliers and heteroscedasticity in the data, a base-10 logarithm of the speed in meters per 15 min was used. The points that were zero-speed intervals were considered to be NA for log-transformed and omitted from the analyses. Explanatory variables included key spatiotemporal factors: land type habitat (forest vs. human habitat and beaches), tourist season (low vs. high), and time of day (late night vs. morning, midday, and evening). To account for repeated measures and individual variability in movement behavior, dog ID, which varied across different tourist seasons, was incorporated as a random effect.

- Dogs’ habitat and land type preferences (b)

All locations of each dog were cross-classified by land type habitats, ownership status, tourist season, and time of day. Land type was the most critical dependent variable due to differences in the risk of human–dog contact and in defining the level of difficulty in future dog approaches for dog disease control programs. The outcome variable was treated as a multinomial variable (forest vs. human habitat and beaches) under testable independence of tourist season, time of day, and random effect of dog using Bayesian multinomial mixed logistic regression by the “brms” package in R software [58]. All data analysis was conducted using R software version 4.5.0, [57].

### 3.9. Ethical Consideration

This study was conducted in compliance with and was approved by the Animal Ethics Committee of Prince of Songkla University, Thailand (Ref.Al102/2024)—Approval Date: 11 September 2024. All work with unowned free-roaming dogs was conducted with the approval of the institutional Animal Ethics Committee and the consent of the zoophiles responsible for unowned dog management.

## 4. Results

### 4.1. Background Information on the Dogs

Background details of the dog samples are shown in Table 1. Overall, fifteen dogs (eight owned and seven unowned) were tracked. Ten dogs were tracked in both low and high tourist seasons. Three dogs were tracked only during the low tourist season, and two dogs were tracked only during the high tourist season. Thus, there were 13 dogs in the low tourist season and 12 dogs in the high tourist season. Most of the samples (12 of 15) were neutered male dogs. We did not have the facility to weigh the dogs. Therefore, sex, neutering status, and weight were not included in the analysis.

### 4.2. Movement Pattern

- Daily distance

On average, the dog’s speed was 55.2 ± 20.1 m per 15 min. The average daily distance was 5302 ± 1928 m. Unowned dogs traveled more than owned dogs, both in the low tourist season (5721 ± 1680 and 5384 ± 2788 m) and the high tourist season (5112 ± 1374 and 4975 ± 1845 m). There was no significant difference in speeds between season and ownership status using ANOVA (*p*-value > 0.05).

- Speed hourly pattern

**Figure 3:**
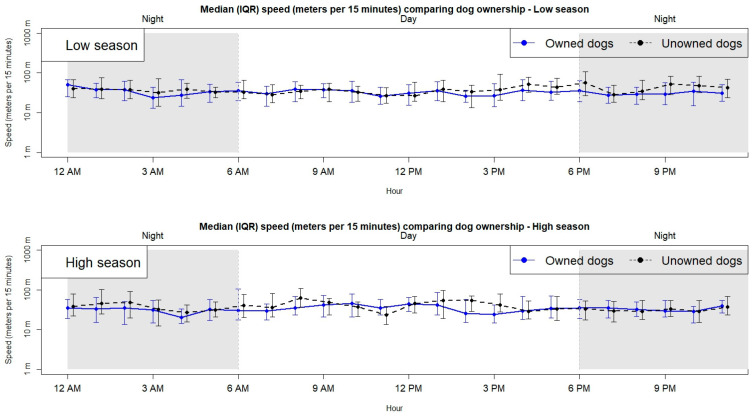
Median and IQR of speed (meters per 15 min), stratified by hour of day, ownership status (owned vs. unowned dogs), and season (low tourist season: June–October; high tourist season: November–May).

Figure 3 visualizes the median speed stratified by ownership status, season, and hour. It compares the median speed between owned (solid line) and unowned (dashed line) dogs in low (upper graph) and high (lower graph) tourist seasons.

- Dogs’ speed by land type.

Notably, no clear diurnal pattern was observed, as the dog remained active (speed > 0 m per 15 min) throughout the 24 h cycle, regardless of whether the dog was owned or unowned. The lowest speeds were recorded in owned dogs at 04:00 a.m. during the high tourist season. The highest speeds were observed in unowned dogs at 01:00 a.m. during high tourist season as well.

**Figure 4:**
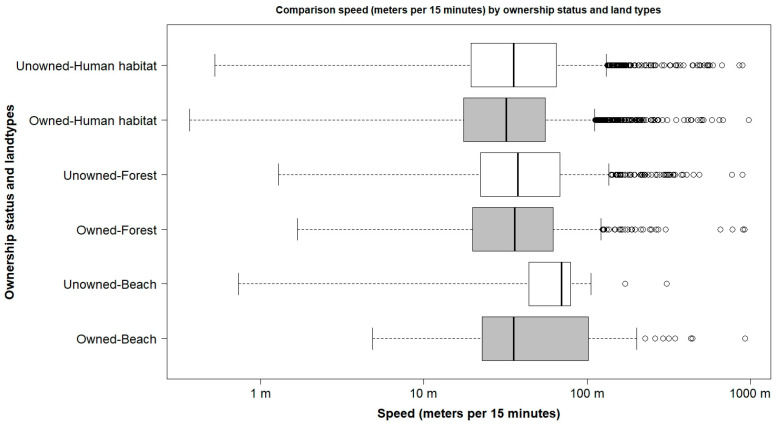
Dogs’ speed (meters per 15 min) comparison between ownership status and the land type habitats.

Figure 4 compares the dog’s speed of owned (gray color) and unowned (white color) dogs, categorized by land type habitat: beaches, forests, and human habitats. Higher speed was observed in unowned dogs at beaches, with a median of 70 m per 15 min (IQR: 44–79 m). In the forest and around human habitat, the difference between the two groups of dogs was relatively small.

- Factors influencing dogs’ speed

Out of 5511 transient (between two sequential points) speeds, 885 were zero and transformed to missing.

The linear mixed model further analyzed the effect of land type on the dogs’ movement speed, adjusting for the impact of tourist season and time of day. The results are summarized in Table 2. The land type variable remained a significant predictor of speed’s movement.

For the sensitivity analysis, instead of treating zero as missing, we repeated the analysis using the mean speed. The regression results remained unchanged (Table S2).

On beaches, on average, dogs ran 10^0.17^ = 1.46 times (an increase of 46%) faster than in the forest, whereas the speed was reduced by 10^−0.10^ = 0.79 (a decrease of 21%) around the human habitat. There was no evidence of the effect of other independent variables.

### 4.3. Dogs’ Habitat and Land Type Preferences

- Overview of dogs’ habitat and distribution

Table 3 tabulates the distribution of dogs appearing by ownership and tourist season. Human habitats were the primary locations for most dogs (75%), with over 80% owned and more than 65% unowned. However, the ratios of dogs appearing in human habitats compared to forests were higher among owned dogs (8:1.5) than unowned dogs (6.5:3). Beaches were the land types where dogs were least frequently appearing.

- Factors contributing to dogs’ appearance in land type habitats

We predicted the presence of dogs in different land types, adjusting for possible confounding effects of other independent variables, using Bayesian multinomial mixed logistic regression. The results are presented in Table 4, which is divided into columns for beaches and human habitat outcomes. The middle column displays the effect of the independent variable on the presence of dogs on beaches. The right column examines the influence of these dogs on human habitats. Both outcomes use the forest as the reference outcome.

Dogs on the island were most likely to appear in human habitats, but very rarely on beaches relative to forests, with ORs (95% CI) of 16.90 (1.92–156.49) and 0.01 (0.00–0.26), respectively. The time of day was a significant factor influencing dogs in different times and land types. The dogs were more likely to be on beaches during midday, while they were more likely to be in human habitats during the evening relative to forests in the late night, with ORs (95% CI) of 1.85 (1.00–3.55) and 1.47 (1.18–1.82), respectively. The tourist season factors did not clearly influence dogs across land types in the model.

## 5. Discussion

In this study, we describe the pattern of movement and habitat of free-roaming dogs on the 2 km^2^ Lipe Island, a terrain consisting of a mixture of forests, human habitats, and beaches. Dogs traveled around 5000 m per day, with the fastest speed on the beaches and the slowest in human habitats. The dogs mainly stayed in human habitat areas. The dogs appeared on beaches during midday, while they were more likely in human habitats during the evening. These findings were consistent across months.

The daily distance traveled by dogs on Lipe Island was greater than that reported in previous studies in other geographical regions [33,59], which could be due to differences in terrain. The island’s landscape is a small area without territorial barriers, such as roads and rivers. The dogs also did not demonstrate any diurnal pattern. Local tropical temperatures may contribute to shortened sleep duration during the night [36]. Lipe Island is also an interesting tourist destination throughout the year. The tourists’ nightlife generates artificial light and noise, which could disrupt dogs’ sleep–awake cycle through the cortisol hormone and melatonin pathway [60]. These reasons may explain why our sample dogs had longer wandering distances and did not exhibit a diurnal activity pattern.

Our dog samples traveled at a high speed on beaches and at a slower speed in human habitats. The beaches on Lipe Island feature long, narrow stretches with minimal development and are not crowded; dogs are also allowed to run freely. In forests and shrublands, the speeds were slower because vegetation obstructs dogs’ movement. In human habitats, the crowding of people reduces dog activity behavior in areas of human disturbance [24]. High dog activity in areas of human habitation increases interactions between humans and dogs, leading to more exposure to dogs and a higher likelihood of transmitting zoonotic diseases [61,62].

In our study, the dog samples were mostly found in human habitats. Since dogs are companion domestic animals that typically reside in close proximity to human society for their biological needs, including food and a sense of safety [30,40], they are more commonly found in human habitats than in other land types. Conversely, the dog samples’ coordinates showed they were least likely to be found along beaches; this is due to the smaller area size of beaches compared to other land types.

Our study revealed that the presence of dogs in human habitats in the evening is likely driven by food-seeking or returning home [63]. However, we could not find why dogs prefer to be on beaches during midday. There may be biological aspects we need to investigate further.

The island has a high dog population density compared to previous studies [25,38]. We consider that this island is vulnerable to dog-related diseases, such as rabies [5,9], which may affect human health. Firstly, both dogs and humans have high population densities on this island. Secondly, there is a substantial number of roaming dogs that travel long distances relative to the island’s size (5000 m per day in the 2 km^2^ island). If the rabies virus were to land on the island and infect a dog, the disease could spread very rapidly. Therefore, the dog immunization coverage must be maintained at a sufficiently high level throughout the entire year. Additionally, unowned dogs that live in forest areas can easily miss the immunization. Dog shelters, enforcing dog confinement, and collaborating with local zoophiles are thus recommended. Finally, the fundamental problem of high dog density resulting from owner negligence requires improvements in dog registration.

In our study, we did not consider the ownership status, gender, or neutering status of dog behavior. However, the unowned dogs in our study received care from zoophiles, who provide food, water, and basic health support, while owned dogs received similar care from their owners. This shared availability of attention is likely to reduce behavioral differences between the two groups [64]. In addition, although almost all dogs in our study were neutered male dogs, a previous study revealed that neutering status had no impact on home range in male free-roaming dogs [65]. Our sample size was small (15 dogs), which might limit hypothesis testing. However, the population size of these dogs on the island was limited to approximately 120. Thus, the small sample size was not a major problem.

Most of the previous studies on dogs’ movement were conducted in the human habitat, either rural or urban landscapes [30,31,32,38,39,42,55,59]. Our study provides new insights from a relatively small tourist island where dogs are almost entirely free-roaming. However, our data have limitations, including possible bias in dog selection (because their difficult-to-fit GPS collars were self-excluded from the study), baseline behavior assessment before collar use, the precision of the GPS devices, a relatively short duration of dog monitoring, and data losses due to technical reasons. The findings must be interpreted with caution.

## 6. Conclusions and Recommendations

In conclusion, free-roaming dogs on this high-dog-density tourist island exhibit a substantial daily travel distance that shows no significant diurnal or monthly variation. However, land type preferences vary by time of day.

Our findings underscore the importance of addressing the fundamental problems of high dog density and owner negligence, as well as the need to improve dog registration and other measures to reduce free-roaming dog activity and maintain a high level of rabies immunization throughout the year.

## Figures and Tables

**Table 1:** Characteristics of dogs and their tracked times in the study of the movement dynamics and habitat use of free-roaming dogs on Lipe Island (N = 15).

No	ID	Round	Sex	Age (Years)	Tracking Time (Hours)		Total Fixed (Points)	
					Low Season	High Season	Low Season	High Season
1	Owned-01	1, 3	Male	>5	96	85	319	268
2	Owned-03	1, 4	Female	>5	96	78	306	171
3	Owned-04	1, 4	Male	>5	96	78	281	233
4	Owned-05	1, 3	Male	1–5	40	83	44	245
5	Owned-06	1, -	Male	1–5	96	-	271	-
6	Owned-07	1, 3	Male	>5	96	85	296	299
7	Owned-08	-, 4	Male	>5	-	78	-	221
8	Owned-09	1, -	Female	1–5	96	-	192	-
9	Unowned-02	2, 4	Male	>5	83	77	228	225
10	Unowned-03	2, 3	Male	>5	82	85	83	219
11	Unowned-04	2, 3	Male	>5	79	80	89	141
12	Unowned-05	2, -	Male	1–5	89	-	124	-
13	Unowned-06	2, 4	Male	>5	80	78	239	251
14	Unowned-07	2, 3	Male	>5	83	85	303	296
15	Unowned-08	-, 4	Male	>5	-	78	-	167

**Table 2:** Linear mixed model predicting the dogs’ speed on different land types, adjusted for time of day and tourist season (** = *p* < 0.005).

**Outcome: log10 of Speed (Meters per 15 min)**			
**Predictors**	**Estimates**	**CI**	***p*-Value**
(Intercept)	1.61	1.56, 1.66	<0.001
Land type habitats			
- Forests (Ref.)			
- Beaches	0.17	0.06, 0.27	0.001 **
- Human habitat	−0.10	−0.13, −0.07	<0.001 **
Tourist season			
- Low tourist season (Ref.)			
- High tourist season	0.00	−0.02, 0.03	0.835
Time of day			
- Late night (22:00–03:59) (Ref.)			
- Morning (04:00–09:59)	−0.02	−0.06, 0.01	0.155
- Midday (10:00–15:59)	−0.01	−0.05, 0.02	0.436
- Evening (16:00–21:59)	−0.00	−0.04, 0.03	0.786

**Table 3:** The number of dogs’ coordinates distributed among land type habitats, separated by tourist season and ownership status.

Land Type Habitats	Owned Dogs		Unowned Dogs	
	Low Season (%col)	High Season (%col)	Low Season (%col)	High Season (%col)
Human habitat	1380 (80.75)	1179 (82.05)	722 (67.73)	845 (65.05)
Forests	301 (17.61)	225 (15.66)	325 (30.49)	442 (34.03)
Beaches	28 (1.64)	33 (2.30)	19 (1.78)	12 (0.92)
Total points	1709 (100)	1437 (100)	1066 (100)	1299 (100)

**Table 4:** Bayesian multinomial mixed logistic regression predicting the presence of dogs in different land types (using forest as the reference level) adjusted by possible confounders.

Predictor	Using Forest as a Reference	
	Outcome as Beaches; OR (95%CI)	Outcome as Human Habitats; OR (95%CI)
Intercept	0.02 (0.00, 0.12)	8.17 (1.89, 32.79)
Tourist season		
- Low tourist season (Ref.)		
- High tourist season	0.96 (0.60, 1.57)	1.10 (0.94, 1.31)
Time of day		
- Late night (22:00–03:59) (Ref.)		
- Morning (04:00–09:59)	1.14 (0.59, 2.25)	0.92 (0.74, 1.14)
- Midday (10:00–15:59)	1.85 (1.02, 3.46)	0.91 (0.72, 1.15)
- Evening (16:00–21:59)	1.52 (0.81, 2.87)	1.47 (1.17, 1.84)

## Data Availability

The original contributions presented in this study are included in the article/Supplementary Materials. Further inquiries can be directed to the corresponding author.

## Supplementary Materials

The following supporting information can be downloaded at https://www.mdpi.com/article/10.3390/vetsci12121181/s1, Figure S1: Figure showing a dog fitted with a collar containing a Catlog Gen 2 GPS device; Figure S2: Flow of the data cleaning process; Table S1: Summary of daily distances observed in dogs across multiple studies; Table S2: Linear mixed model predicting the dogs’ speed on different land types, adjusted for other variables, zero-speed values were replaced by the speed’s mean (** = *p* < 0.005).
