# Supplementary material for: Movement Dynamics and Habitat Use of Owned and Unowned Free-Roaming Dogs on a Two-Square-Kilometer Tourist Island in Southern Thailand
Source: Vet Sci. 2025 Dec 10;12(12):1181. doi: 10.3390/vetsci12121181 (PMC12737669; doi:10.3390/vetsci12121181)
Supplement: Supplementary file 1 [file vetsci-12-01181-s001.zip › Table S2.pdf]

Table S2**:** Linear mixed model predicting the dogs' speed on different land types, adjusted for other variables, zero-speed values were replaced by the speed's mean (* * = $p < 0.005$).

**Outcome: log10 of speed (meters per 15 minutes)**			
**Predictors**	**Estimates**	**CI**	**p-value**
(Intercept)	-1.37	-1.41, -1.33	**<0.001**
Land type habitats			
- Forest or shrub (Ref.)			
- Beach	0.12	0.04, 0.21	**0.003**
- Human habitat	-0.08	-0.11, -0.06	**<0.001**
Tourist season			
- Low tourist season (Ref.)			
- High tourist season	-0.00	-0.02, 0.02	0.909
Time of day			
- Late night (22:00-03:59) (Ref.)			
- Morning (04:00-09:59)	-0.01	-0.04, 0.02	0.424
- Midday (10:00-15:59)	0.01	-0.02, 0.03	0.706

**Outcome: log10 of speed (meters per 15 minutes)**			
**Predictors**	**Estimates**	**CI**	**p-value**
- Evening (16:00-21:59)	-0.01	-0.03, 0.02	0.646
