# Supplementary material for: Movement Dynamics and Habitat Use of Owned and Unowned Free-Roaming Dogs on a Two-Square-Kilometer Tourist Island in Southern Thailand
Source: Vet Sci. 2025 Dec 10;12(12):1181. doi: 10.3390/vetsci12121181 (PMC12737669; doi:10.3390/vetsci12121181)

Figure S2**:** Flow of the data cleaning process

A total of 7,038 points were exported from the GPS devices; we excluded 169 points located outside the island, 50 points recorded before the reference starting time, 9 points with HDOP > 5, and 1,328 points recorded after the reference ending time.

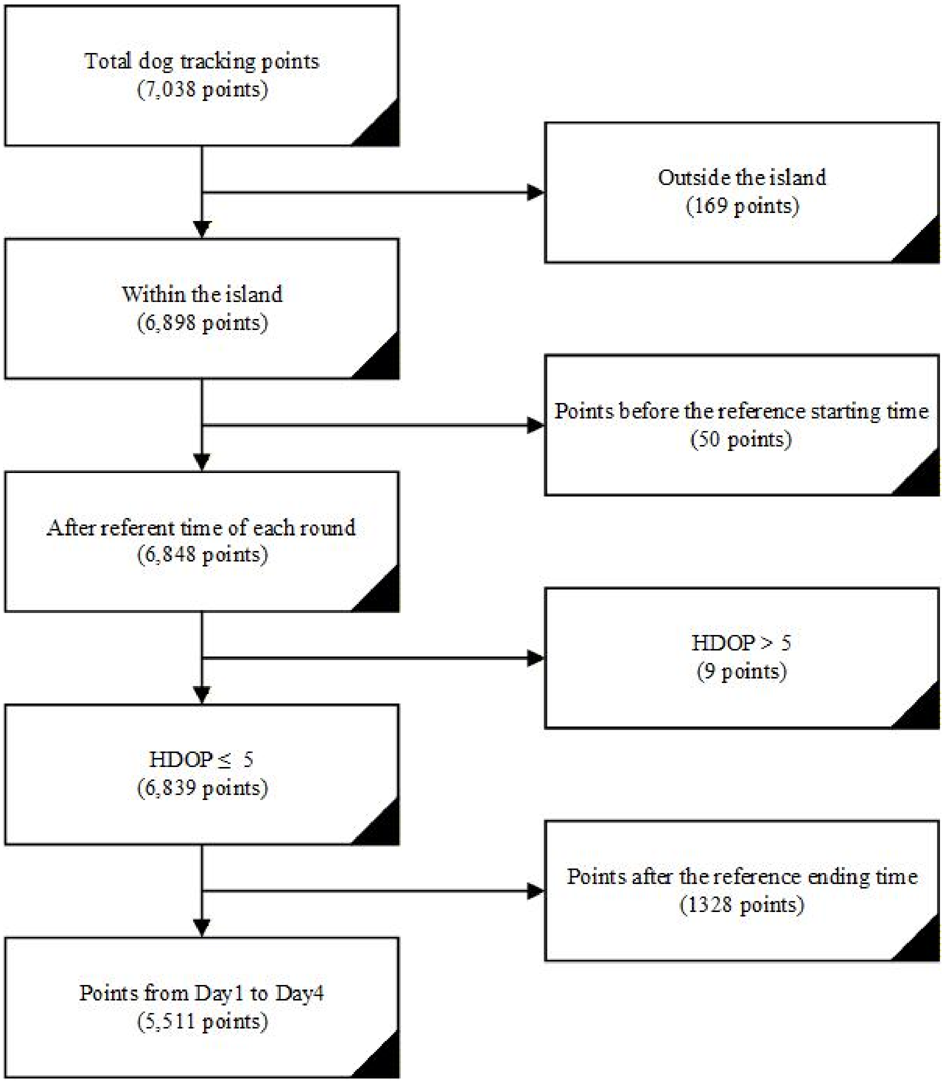

Supplement: Supplementary file 1 [file vetsci-12-01181-s001.zip › Figure S2.pdf]
