# Supplementary material for: Movement Dynamics and Habitat Use of Owned and Unowned Free-Roaming Dogs on a Two-Square-Kilometer Tourist Island in Southern Thailand
Source: Vet Sci. 2025 Dec 10;12(12):1181. doi: 10.3390/vetsci12121181 (PMC12737669; doi:10.3390/vetsci12121181)
Supplement: Supplementary file 1 [file vetsci-12-01181-s001.zip › Table S1.pdf]

Table S1:Summary of daily distances observed in dogs across multiple studies

Year-Authors	Study setting	Study periods	Dog samples	Results	
				Daily distances	Peak movement time
Mutwiri, Muigai et al. (2023)	Western part of Kenya	GPS tracking, every minute for 5 days	73 homesteads owning free-roaming dogs	Ranges: 2 - 24.5 km (median 13.5 km)	Not mentioned
Pérez, Conte et al. (2018)	In a remote Patagonian city surrounded by a grassland's rural ecosystem in Puerto Natales, Chile.	GPS tracking, every 2 minutes for 3 - 11 days (3 - 4 days per session and 1–3 sessions per dog)	86 male-owned free-roaming dogs	Maximum distance ranges from 0.1 - 10.4 km (average 1.1 km) after removing outliers, which range from 0.1 - 3.1 km (average 0.84 km)	More fixed detection between 12:00–16:00 h
Kuo, Chang et al. (2025)	Yangmingshan Taiwan, a mountainous region located in the suburbs of Taipei City and New Taipei City	GPS tracking, every 30 minutes, with mean tracking periods of 137 ± 83 days in owned dogs and 101 ± 38 days in unowned dogs.	12 owned dogs and 10 unowned dogs	Mean ± SE = 1.51 ± 0.55 km in owned dogs and 1.69 ± 1.06 km in unowned dogs	Unowned dogs were typically found closer to roads or anthropogenic areas on weekends, but there was no difference between daytime and nighttime observations.
Sparkes, Körtner et al. (2022)	Peri-urban sites in north-east New South Wales and south-east	GPS tracking, every 15 minutes, between September 2013 and August 2014	43 free-roaming owned dogs	Ranges from 0.25 - 4.81 km (mean±SD = 1.95 ± 1.09 km)	0700 - 1000 and 1600 - 1900 hrs.

Year-Authors	Study setting	Study periods	Dog samples	Results	
				Daily distances	Peak movement time
	Queensland, Australia	in north-east NSW, and between December 2014 and February 2015 in south-east QLD.			
Wada, Mazlan et al. (2025)	Malaysia	Field survey, from March 22, 2022, to October 6, 2022	Free-roaming dogs (including family-owned dogs, community-owned dogs, and unowned/stray dogs)		Observation during pilot study founded that dog more active in morning and evening.
Carvalho, Rosalino et al. (2019)	In rural areas near the forest, southeastern Brazil	714 camera-trap days (17 cameras-trap running for 42 days) during July 2006 to February 2007 and July 2009 to February 2010	Domestic dogs	Not mentioned	From a diurnal activity pattern to a cathemeral activity pattern

REFERENCES

1. Mutwiri, T.; Muigai, A.W.T.; Magambo, J.; Mulinge, E.; Gitau, L.; Muinde, P.; Bettridge, J.M.; Rogan, M.; Fèvre, E.M.; Falzon, L.C. The potential role of roaming dogs in establishing a geographically novel life cycle of taeniids (Echinococcus spp. and Taenia spp.) in a non-endemic area. Vet. Parasitol. Reg. Stud. Rep. 2023, 38, 100829. https://doi.org/10.1016/j.vprsr.2022.100829.

2. Pérez, G.E.; Conte, A.; Garde, E.J.; Messori, S.; Vanderstichel, R.; Serpell, J. Movement and home range of owned free-roaming male dogs in Puerto Natales, Chile. Appl. Anim. Behav. Sci. 2018, 205, 74–82. https://doi.org/10.1016/j.applanim.2018.05.022.

3. Kuo, T.-H.; Chang, G.-M.; Yu, P.-H.; Chen, W.-H.; Yen, S.-C. The difference in roaming behavior between owned and unowned dogs in a satoyama landscape area. Appl. Anim. Behav. Sci. 2025, 283, 106521. https://doi.org/10.1016/j.applanim.2025.106521.

4. Sparkes, J.; Körtner, G.; Ballard, G.; Fleming, P.J.S. Spatial and temporal activity patterns of owned, free-roaming dogs in coastal eastern Australia. Prev. Vet. Med. 2022, 204, 105641. https://doi.org/10.1016/j.prevetmed.2022.105641.

5. Wada, Y.A.; Mazlan, M.; Noordin, M.M.; Mohd-Lila, M.A.; Fong, L.S.; Ramanoon, S.Z.; Zahli, N.I.U. Free-roaming dog population and density in Klang Valley, Peninsular Malaysia: A comparative enumeration method for improved management and rabies control. Prev. Vet. Med. 2025, 240, 106536. https://doi.org/10.1016/j.prevetmed.2025.106536.

6. Carvalho, W.D.; Rosalino, L.M.; Godoy, M.S.A.M.; Giorgete, M.F.; Adania, C.H.; Esbérard, C.E.L. Temporal activity of rural free-ranging dogs: Implications for the predator and prey species in the Brazilian Atlantic Forest. NeoBiota 2019, 45, 55–74. https://doi.org/10.3897/neobiota.45.30645.
