# Supplementary figures and images for: Movement Dynamics and Habitat Use of Owned and Unowned Free-Roaming Dogs on a Two-Square-Kilometer Tourist Island in Southern Thailand
Source: Vet Sci. 2025 Dec 10;12(12):1181. doi: 10.3390/vetsci12121181 (PMC12737669; doi:10.3390/vetsci12121181)

Figure S1: Figure showing a dog fitted with a collar containing a Catlog Gen 2 GPS device

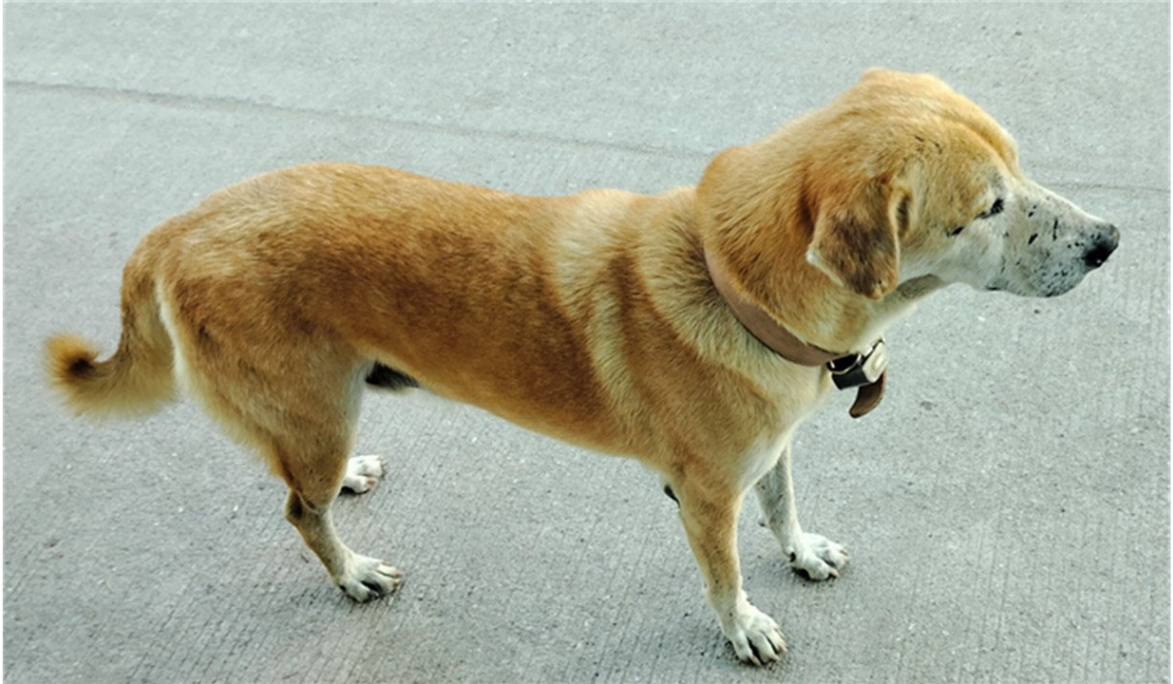

Supplement: Supplementary file 1 [file vetsci-12-01181-s001.zip › Figure S1.pdf]
